# Acute neuroinflammation induces AIS structural plasticity in a NOX2-dependent manner

**DOI:** 10.1186/s12974-017-0889-3

**Published:** 2017-06-08

**Authors:** S. D. Benusa, N. M. George, B. A. Sword, G. H. DeVries, J. L. Dupree

**Affiliations:** 1 Department of Anatomy and Neurobiology, Virginia Commonwealth University, PO Box 980709, , 1101 East Marshall Street, Richmond, VA 23298 USA; 2 Neuroscience Curriculum, Virginia Commonwealth University, Richmond, VA 23298 USA; 3 Neuroscience Graduate Program, University of Colorado, Denver, CO 80204 USA; 4 Research Service 151, Hunter Holmes McGuire Veterans Affairs Medical Center, Department of Veterans Affairs, 1201 Broad Rock Blvd, Richmond, VA 23249 USA

**Keywords:** Axon initial segment, NOX2, Calpain, Reactive oxygen species, Neuroinflammation, Microglia

## Abstract

**Background:**

Chronic microglia-mediated inflammation and oxidative stress are well-characterized underlying factors in neurodegenerative disease, whereby reactive inflammatory microglia enhance ROS production and impact neuronal integrity. Recently, it has been shown that during chronic inflammation, neuronal integrity is compromised through targeted disruption of the axon initial segment (AIS), the axonal domain critical for action potential initiation. AIS disruption was associated with contact by reactive inflammatory microglia which wrap around the AIS, increasing association with disease progression. While it is clear that chronic microglial inflammation and enhanced ROS production impact neuronal integrity, little is known about how acute microglial inflammation influences AIS stability. Here, we demonstrate that acute neuroinflammation induces AIS structural plasticity in a ROS-mediated and calpain-dependent manner.

**Methods:**

C57BL/6J and NOX2^−/−^ mice were given a single injection of lipopolysaccharide (LPS; 5 mg/kg) or vehicle (0.9% saline, 10 mL/kg) and analyzed at 6 h–2 weeks post-injection. Anti-inflammatory Didox (250 mg/kg) or vehicle (0.9% saline, 10 mL/kg) was administered beginning 24 h post-LPS injection and continued for 5 days; animals were analyzed 1 week post-injection. Microglial inflammation was assessed using immunohistochemistry (IHC) and RT-qPCR, and AIS integrity was quantitatively analyzed using ankyrinG immunolabeling. Data were statistically compared by one-way or two-way ANOVA where mean differences were significant as assessed using Tukey’s post hoc analysis.

**Results:**

LPS-induced neuroinflammation, characterized by enhanced microglial inflammation and increased expression of ROS-producing enzymes, altered AIS protein clustering. Importantly, inflammation-induced AIS changes were reversed following resolution of microglial inflammation. Modulation of the inflammatory response using anti-inflammatory Didox, even after significant AIS disruption occurred, increased the rate of AIS recovery. qPCR and IHC analysis revealed that expression of microglial NOX2, a ROS-producing enzyme, was significantly increased correlating with AIS disruption. Furthermore, ablation of NOX2 prevented inflammation-induced AIS plasticity, suggesting that ROS drive AIS structural plasticity.

**Conclusions:**

In the presence of acute microglial inflammation, the AIS undergoes an adaptive change that is capable of spontaneous recovery. Moreover, recovery can be therapeutically accelerated. Together, these findings underscore the dynamic capabilities of this domain in the presence of a pathological insult and provide evidence that the AIS is a viable therapeutic target.

## Background

The axon initial segment (AIS) is a highly specialized axonal domain responsible for action potential initiation and modulation [1]. The AIS is characterized by a unique assembly of cytoskeletal and scaffold proteins [2] and densely packed voltage-gated ion channels, which are recruited to and clustered at the AIS via the scaffolding protein ankyrinG (ankG) [3]. ankG is considered the “master organizer” of the AIS and is essential for AIS function [4, 5]. Accumulating evidence suggests that the AIS is a dynamic domain capable of structural plasticity, undergoing changes in length [6], location [2, 7, 8], and ion channel clustering [9, 10] in response to neuronal pathology and altered activity.

AIS plasticity is characterized by the relocation of cytoskeletal-associated proteins such as ankG, βIV spectrin, neurofascin, and voltage-gated sodium (NaV) channels [2, 7, 11–13]. Although plasticity can be triggered by both pathologic and non-pathologic stimuli, the mechanisms and cell types that drive plasticity remain largely unknown. Schafer et al. [14] were the first to implicate the calcium-dependent protease calpain as a mediator of AIS plasticity with recent studies confirming these findings [10, 15]. Consistent with calpain activation, Evans et al. [8] reported that AIS plasticity is triggered by calcium channel activation with downstream activation of calcineurin. Recently, it has also been shown that microglia may influence neuronal activity through specific association with the AIS [16]. Microglia-AIS contact was found to occur early in development and persist throughout adulthood in the uninjured brain [16] as well as during chronic inflammation present in an animal model of multiple sclerosis known as experimental autoimmune encephalomyelitis (EAE) [17], suggesting an important interaction that may influence AIS integrity.

Microglia, the resident immune cells of the central nervous system (CNS), are dynamic cells that survey, respond, and shape neuronal networks through neuronal contact and synaptic pruning [18–21]. Microglia are critical for maintaining tissue homeostasis in the CNS, rapidly activating and eliminating pathogens and cellular debris in response to infection or insult [22–24]. Upon activation, microglia display an enhanced pro-inflammatory response and a dampened resolving phenotype [25–27]. This is typified by increased expression of inflammatory mediators such as tumor necrosis factor alpha (Tnf-α), cyclooxygenase-2 (COX-2), and NADPH oxidase 2 (NOX2), elevated production of reactive oxygen species (ROS), and reduced expression of resolving factors such as transforming growth factor beta (TGF-β), mannose receptor, C type 1 (Mrc1), and resistin-like beta (Fizz-1) [28–30]. Though reactive microglia play an important role in pathogen clearance and CNS homeostasis, amplified ROS production or aberrant activation of the inflammatory phenotype has been implicated in a number of neuronal pathologies [31–35] where AIS disruption is observed [14, 16, 17, 36, 37]. Previous studies from our lab demonstrated that chronic neuroinflammation in EAE resulted in changes in AIS length and protein clustering and this disruption corresponded with increased microglial reactivity and production of pro-inflammatory factors [17]. Furthermore, AIS disruption corresponded with increased contact between reactive microglia and the AIS, suggesting that in a chronic inflammatory environment, pro-inflammatory microglia may drive AIS disruption [17].

The microglial inflammatory response is amplified by the enzyme NOX2, which is responsible for the microglial respiratory burst and extracellular production of ROS [38]. NOX2 activity has been implicated in the chronic activation of microglia and its deleterious effects both through the production of extracellular ROS and through amplification of the pro-inflammatory response [39–41]. Inhibition of NOX2 reduced microglial ROS production and reduced microglia-mediated neurotoxicity [40, 42, 43]. Here, we investigate the role of microglial inflammation and the ROS-producing enzyme NOX2 on AIS integrity. Using a lipopolysaccharide (LPS)-induced model of neuroinflammation, we demonstrate that in the presence of acute microglial inflammation, AIS ankG clustering is disrupted and upon resolution of inflammation, AIS changes are reversed. Furthermore, ablation of NOX2 preserved AIS integrity. These data underscore the dynamic capabilities of the AIS in the presence of a pathological insult.

## Methods

### Animals

Six- to eight-week-old C57BL/6J mice and NOX2-deficient (B6.129S-Cybb^tm1Din^/J, NOX2^−/−^) mouse breeding pairs were purchased from Jackson Laboratories (Bar Harbor, ME). All animals were maintained in the AAALAC-accredited McGuire Veterans Affairs Medical Center (VAMC) vivarium with access to food and drink ad libitum. NOX2^−/−^ mice have a targeted mutation of the 91-kD subunit of the oxidase cytochrome b and lack phagocyte superoxide production [44]. NOX2^−/−^ mice are maintained on a C57BL/6J background; therefore, age-matched C57BL/6J mice (NOX2^+/+^) were used as controls. All procedures were conducted in accordance with the National Institutes of Health guidelines for the care and use of laboratory animals and were approved by the McGuire VAMC Institutional Animal Care and Use Committee.

### LPS treatment

Lipopolysaccharide (LPS; O111:B4, lot: 2728527) was purchased from Calbiochem (San Diego, CA). Female C57BL/6J and NOX2^−/−^ mice (8–12 weeks) were given a single intraperitoneal (IP) injection of LPS (5 mg/kg, 10 mL/kg) or vehicle (0.9% saline). The LPS dose was based on the previously established neuroinflammation model [45, 46] where peripheral inflammation rapidly transfers to the brain, resulting in elevated microglial cytokine and ROS production [45]. Saline- and LPS-treated mice were analyzed at 6 h, 24 h, 3 days, 1 week, and 2 weeks post-injection to assess the effects of microglial reactivity and AIS integrity throughout the course of neuroinflammation.

### Didox administration

Didox (3,4-dihydroxybenzohydroxamic acid) was obtained from Molecules for Health, Inc. (Richmond, VA). Didox, a ribonucleotide reductase inhibitor and free radical scavenger, is a multifunctional compound that inhibits DNA replication, suppresses NF-κB activation, reduces oxidative injury, and attenuates microglia/macrophage production of inflammatory cytokines and ROS-producing enzymes [47–49]. Based on previous studies [17, 50], Didox (250 mg/kg solubilized in 0.9% saline) or vehicle (0.9% saline, 10 mL/kg) was administered intraperitoneally beginning at 24 h post-LPS injection and continued for 6 days. Animals were taken for analysis 1 week post-LPS injection.

### Calpain inhibitor administration

Calpeptin was obtained from Calbiochem (San Diego, CA). Calpeptin is a cell-permeable inhibitor of calcium-activated proteases calpain-1 and calpain-2, which have been implicated in targeted cleavage of AIS proteins and alterations in AIS structure [9, 10, 14]. Based on previous studies [51–53], Calpeptin (50 μg/kg) or vehicle (0.1% dimethyl sulfoxide in saline, 10 mL/kg) was administered subcutaneously 30 min prior to injection of LPS (5 mg/kg, 10 mL/kg, IP) or vehicle (0.9% saline, 10 mL/kg, IP). On days 1 and 2 post-LPS injection, mice received a second and third dose of Calpeptin (calpain inhibitor), respectively. Vehicle-, LPS + vehicle-, or LPS + Calpeptin-treated mice were analyzed at 3 days post-LPS injection to assess the effects of calpain activity on AIS integrity.

### Immunohistochemistry

Animals were deeply anesthetized using 0.016 mL/g body weight of a 2.5% solution of avertin (2,2,2 tribromoethanol, Sigma-Aldrich, St. Louis, MO) in 0.9% saline (Sigma-Aldrich, St. Louis, MO) and transcardially perfused with 4% paraformaldehyde (Ted Pella, Redding, CA). Following perfusion, brains were removed and immersed in 0.1 M PBS containing 30% sucrose for 48 h, frozen in OCT compound (Sakura, Netherlands), and serially sectioned into 40-μm-thick coronal sections stored at −80 °C and immunolabeled as previously described [17] using the following antibodies: mouse monoclonal anti-ankyrinG (ankG; NeuroMab, Davis, CA; N106/36; 1:500), rabbit polyclonal anti-Iba-1 (Wako Chemicals, Richmond, VA; 019-19741; 1:1,000), mouse monoclonal anti-NeuN (Millipore, Billerica, MA; MAB377; 1:1000), mouse monoclonal anti-gp91-phox (Santa Cruz, Dallas, TX; sc-130543; 1:500), mouse monoclonal anti-NaV1.6 (NaV1.6; NeuroMab, Davis, CA; K87A/10; 1:200). All secondary antibodies were obtained from Invitrogen Life Technologies (Grand Island, NY; Alexa™ Fluor) and used at a dilution of 1:500.

### Imaging and analysis

Imaging was performed on a Zeiss LSM 710 confocal laser scanning microscope (Carl Zeiss Microscopy, LLC, Thornwood, NY) housed in the VCU Department of Anatomy and Neurobiology Microscopy Facility. For AIS number analysis, images were collected as previously described [17]. Briefly, confocal z-stacks spanning an optical thickness of 25 μm, using a pinhole of 1 Airy disc unit and Nyquist sampling (optical slice thickness, 0.48 μm), were collected from neocortical layer V for each of six sections (spanning 1.1 mm anterior to the bregma to 2.5 mm posterior to the bregma) per mouse resulting in 12 images per animal (*n* = 4–6 animals per treatment group). Images were then processed and analyzed using FIJI (NIH ImageJ software). Settings were optimized by comparing manual AIS tracings (previously described by [17]) and FIJI automated counts; no significant difference was found between methods (data not shown). Once established, settings remained constant throughout analysis. Thresholds of maximum intensity projections of ankG labeling were automatically set using the Otsu threshold method [54], and AISs were quantified using the “Analyze Particles” plugin (FIJI) (size 0–infinity μm^2^; circularity 0–0.5; objects touching edges excluded). ankG-positive structures measuring <10 μm were excluded from analysis consistent with previous studies [17, 36, 37].

For analysis of microglial NOX2 immunoreactivity, confocal z-stacks spanning an optical thickness of 25 μm were collected from neocortical layer V for each of six sections (spanning 1.1 mm anterior to the bregma to 2.5 mm posterior to the bregma) per mouse (*n* = 3 animals per treatment group). Images were blinded, and NOX2 immunoreactivity in Iba-1^+^ cells was quantified using Volocity™ 3D Image Analysis Software version 6.3 allowing 3D confirmation of double immunolabeling in each Iba-1^+^ cell. The total number of microglia and the number of NOX2^+^ microglia were counted manually for each double-immunolabeled z-stack. Data are presented as the percent of NOX2^+^ microglia (Iba-1^+^) per field of view.

For neuronal nuclei analysis (NeuN labeling), four confocal images per mouse were collected using a ×20 objective with a numerical aperture of 1.4 and a pinhole of 1 Airy disc unit. Images were processed and analyzed using FIJI (*n* = 3 mice per treatment group). Settings were optimized by comparing manual NeuN counts (previously described [17]) and FIJI automated counts; no significant difference was found between methods (data not shown). Thresholds of maximum intensity projections of NeuN labeling were automatically set using the Otsu threshold method [55], and neuronal nuclei were quantified using the “Analyze Particles” plugin (size 10–150 μm^2^; circularity 0–1; objects touching edges excluded). No differences in NeuN^+^ cell counts were detected among any treatment groups (NOX2^+/+^ Saline, NOX2^+/+^ LPS-injected, NOX2^−/−^ Saline, or NOX2^−/−^ LPS-injected; Table 1).

**Table 1:** Neuronal density and cortical volume measurements from saline or LPS-injected mice

Treatment group	Average NeuN count (% saline ± SEM)		Average cortical volume ± SEM (μm^3^) × 10^3^	
	NOX2^+/+^	NOX2^−/−^	NOX2^+/+^	NOX2^−/−^
Saline	100 ± 3.2	100 ± 7.3	1.4 ± 0.2	1.14 ± 0.3
LPS 6 h	103 ± 5.9	–	1.1 ± 0.2	–
LPS 24 h	98 ± 0.8	109 ± 4.9	1.1 ± 0.1	1.15 ± 0.3
LPS 3 days	108.1 ± 2.5	–	1.4 ± 0.1	–
LPS 1 week	97.7 ± 2.5	110 ± 3.4	1.2 ± 0.1	1.27 ± 0.1
LPS 2 weeks	108 ± 3.9	–	1.5 ± 0.1	–

No significant difference was detected with regard to density of neuronal cell bodies or cortical volume among any of the treatment groups in either NOX2^+/+^ or NOX2^−/−^ mice

Cortical volume analysis was performed using the Cavalieri principle as previously described (modified [55, 56]). Briefly, unbiased stereology was performed using every 15th section from the total sections spanning the cortical region 1.1 mm anterior to the bregma to 2.5 mm posterior to the bregma and analyzed to estimate cortical volume. Each reference space was outlined with a ×2 objective and analyzed using a point-grid analysis, sampling 100% of the regions of interest. Samples were counted in a blind manner and volumes calculated using an Olympus BX51 microscope (Center Valley, PA) and newCAST software (Visiopharm, Hoersholm, Denmark) (*n* = 3–4 mice per treatment group). No differences in cortical volumes were detected among any treatment groups (NOX2^+/+^ Saline, NOX2^+/+^ LPS-injected, NOX2^−/−^ Saline, or NOX2^−/−^ LPS-injected; Table 1).

### Microglia isolation

Adult cortical microglia were isolated using MACS magnetic bead separation (Miltenyi Biotec, San Diego, CA) as described previously [17, 45]. Briefly, saline-treated and LPS-treated mice were deeply anesthetized and transcardially perfused with 50 mL ice-cold PBS. After removal of the meninges, the cerebral cortices of two mice were harvested and pooled per sample (2 mice = 1 *n*) and suspended in Hank’s balanced salt solution (HBSS) without CaCl_2_ and MgCl_2_ (Corning, Corning, NY). A single-cell suspension was prepared using the Miltenyi Neural Tissue Dissociation Kit according to the manufacturer’s instructions. The cells were depleted of myelin by suspension in 3 mL of 30% isotonic Percoll™ (GE Healthcare Life Sciences, Pittsburgh, PA) followed by a 10-min centrifugation at 700 x *g* at 4 °C. The cell pellet was washed in 5 mL HBSS without CaCl_2_ and MgCL_2_, and isolation of microglia was performed with magnetic CD11b microbeads (Miltenyi) and MACS magnetic separator (Miltenyi) according to the manufacturer’s instructions.

### RNA isolation and RT-qPCR analysis

Total RNA was extracted from isolated CD11b^+^ cells or whole cortical tissue using a Qiagen RNeasy mini kit (Qiagen, Germantown, MD) and treated with Ambion DNase I (Invitrogen Life Technologies, Grand Island, NY) (*n* = 3 samples per treatment group). RNA concentrations were determined using a NanoPhotometer (Implen, Los Angeles, CA), and purity was assessed by the ratio of absorbance at 260 and 280 nm (OD_260/280_ > 1.8). Oligo-dT-primed complementary DNAs (cDNAs) were synthesized from 0.25 μg of RNA for each sample using the iScript Reverse Transcription Supermix (Bio-Rad) according to the manufacturer’s guidelines. RT-qPCR reactions with at least two technical replicates per sample were performed on a CFX96 real-time PCR detection system (Bio-Rad) using 1 μL of cDNA, SsoFast Evagreen Supermix (Bio-Rad), and forward and reverse primers (500 nM). Cycling parameters were 1 cycle of 95 °C (5 min), 40 cycles of 95 °C (5 s), and 56 °C (5 s) followed by a melt curve measurement consisting of 5-s 0.5 °C incremental increases from 65 to 95 °C. Relative changes in gene expression were calculated by the 2^−ΔΔCt^ method [57] using cyclophilin A and phosphoglycerate kinase 1 (PGK1) as an endogenous reference gene. Gene-specific primers were designed and checked for specificity using National Center for Biotechnology Information/Primer-BLAST (basic local alignment search tool [58]) (Table 2). Primers were generated by Integrated DNA Technologies (San Diego, CA).

**Table 2:** Oligonucleotide primer sets used for RT-qPCR

Gene	Accession no.	Forward primer	Reverse primer
Tnf-α	NM_013693.1	5′-GCCCACGTCGTAGCAAACCACC-3′	5′-CCCATCGGCTGGCACCACTA-3′
COX-2 (Ptgs2)	NM_009367.1	5′-TTGCTGGCCGGGTTGCTGG-3′	5′-CAGGGAGAAGCGTTTGCGGT-3′
NOX2 (Cybb)	NM_023965.1	5′-GGGAACTGGGCTGTGAATGA-3′	5′-CAGTGCTGACCCAAGGAGTT-3′
Mrc1	NM_008625.2	5′-GGCTGATTACGAGCAGTGGA-3′	5′-CATCACTCCAGGTGAACCCC-3′
Fizz-1 (Retnlb)	NM_020509.3	5′-CAGCTGATGGTCCCAGTGAAT-3′	5′-AGTGGAGGGATAGTTAGCTGG-3′
TGF-β	NM_009367.2	5′-CTCCCCTCCGAAAATGCCA-3′	5′-GTTTTGCAAGCGGAAGACCC-3′
Cyclophilin A	NM_008907.1	5′-CTAGAGGGCATGGATGTGGT-3′	5′-TGACATCCTTCAGTGGCTTG-3′
PGK1	NM_008828.3	5′-ATGCAAAGACTGGCCAAGCTA C-3′	5′-AGCCACAGCCTCAGCATATTTC-3′

### Calpain activity assay

To quantify the levels of calpain activity and to determine the effect of Calpeptin on inhibition of calpain activity, vehicle-, LPS + vehicle-, or LPS + Calpeptin-treated mice were deeply anesthetized and transcardially perfused with 50 mL ice-cold 0.9% saline at 3 days post-LPS injection. Cerebral cortices (10 mg) were harvested and immediately homogenized in ice-cold extraction buffer (Calpain activity kit). Samples were centrifuged for 5 min at 4 °C at 15,000 x *g* to remove insoluble material. Calpain activity was quantified using a fluorometric calpain activity assay kit (ab65308, Abcam, Cambridge, MA) according to the manufacturer’s protocol. All samples were analyzed in triplicate, and calpain activity was measured using a Tecan M1000 PRO microplate reader (Männedorf, Switzerland). Changes in calpain activity were normalized to saline control levels and expressed as relative fluorescent units (RFU).

### Statistical analysis

All graphing and statistical analyses were performed using GraphPad Prism version 6.03 (GraphPad Software, San Diego, CA). Data were analyzed by a one-way or two-way analysis of variance and, where mean differences were significant, assessed using Tukey’s honest significance difference post hoc analysis. Treatment groups were presented as percent of saline control (% Control ± SEM), and *p* < 0.05 was considered statistically significant.

## Results

### LPS-induced inflammation alters AIS protein clustering

**Fig. 1:**
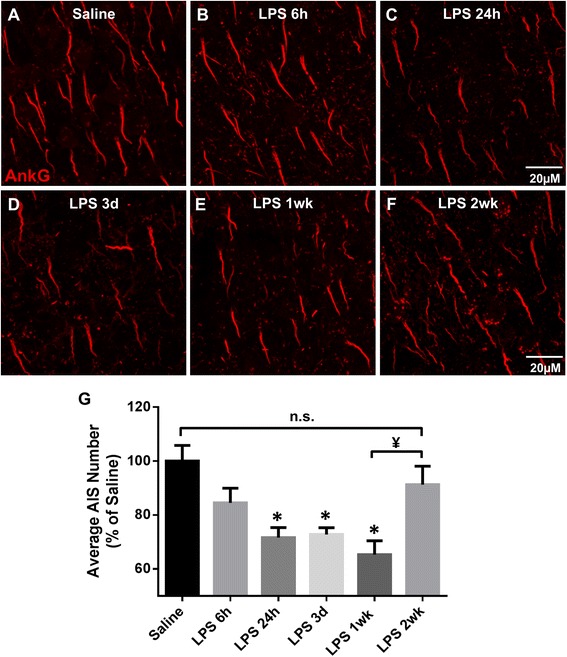
LPS-induced inflammation disrupts AISs. **a**–**f** AISs in layer V of the cerebral cortex identified by ankG immunolabeling in saline- (**a**) and LPS-treated (**b**–**f**) mice. AIS detection is lost with time in LPS-treated mice through 1 week post-injection. However, by 2 weeks post-injection, AIS number returns to control levels, demonstrating reversibility of AIS disruption. *Scale bar* = 20 μM. **g** The mean ± SEM of AISs/FOV in saline- and LPS-treated mice as a percent of saline controls. An *asterisk* indicates significant difference (*p* < 0.05) from saline, and a ¥ indicates a difference between time points

Studies from our lab [17] and others [14, 16, 59] have shown that AIS protein clustering is disrupted during chronic inflammation and disease. To determine if acute neuroinflammation alters AIS integrity, we used a LPS-induced neuroinflammatory model and assessed AIS ankG protein clustering (Fig. 1). AISs were immunolabeled for ankG in saline- and LPS-treated mice at 6 h, 24 h, 3 days, and 1 week post-injection. Disruption of ankG labeling was first observed 24 h post-LPS injection (71.6% ± 3.7, *p* < 0.01) compared to saline controls (100% ± 5.8) (Fig. 1c, f). The number of AISs detected in LPS-injected mice remained significantly decreased at both 3 days and 1 week post-injection (72.7% ± 2.5, *p* < 0.01 and 65.3% ± 5.1, *p* < 0.0001, respectively) compared to saline controls (Fig. 1d–f). AIS disruption as indicated by a loss of ankG immunolabeling was confirmed by immunolabeling for NaV1.6 (data not shown). To determine if AIS disruption was a consequence of neuronal loss or changes in cortical volume, we quantified NeuN immunolabeling and cortical volume and found no difference among saline or LPS groups (Table 1). These data suggest that acute neuroinflammation caused a significant disruption in AIS protein clustering, but altered ankG detection was not associated with neuronal loss.

### AIS disruption is reversible

The AIS is the site of action potential initiation and thus is critical for neuronal function [60]. Studies have shown that the AIS can undergo structural plasticity in development and in response to pathological insults to sustain proper signaling within neuronal networks [2, 6, 61, 62]. To determine in vivo if inflammation-induced AIS disruptions are reversible, we assessed ankG clustering of AISs in saline- and LPS-treated mice 2 weeks post-injection (Fig. 1). ankG immunolabeling revealed that the number of AISs in LPS-treated mice 2 weeks post-injection returned to baseline and was not significantly reduced compared to saline controls (91.3% ± 2.8) (Fig. 1f, g). Furthermore, AISs at 2 weeks post-LPS injection were significantly increased compared to LPS 1 week treated mice (mean difference 25.9% ± 5.7, *p* < 0.01) (Fig. 1e–g). Thus, LPS-induced disruption of AIS ankG clustering is reversible.

### AIS integrity coincides with microglial inflammatory response

**Fig. 2:**
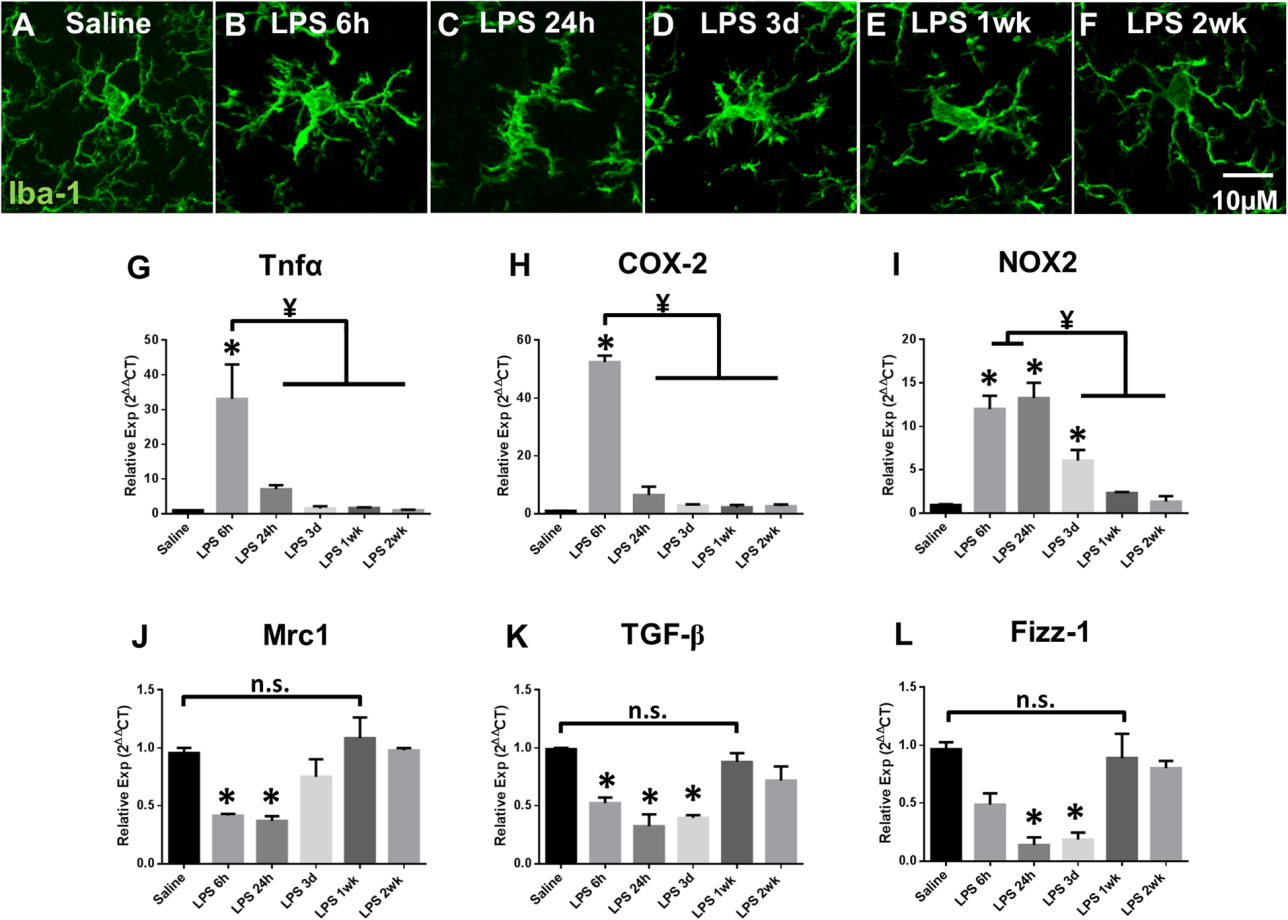
AIS integrity coincides with microglial inflammatory response. **a**–**f** Microglia, visualized by Iba-1 immunolabeling, display a surveying phenotype with long, highly branched processes in saline-treated mice (**a**). Microglia display a highly reactive morphology with retracted, thickened processes at 6 h post-LPS injection (**b**) and maintain this morphology at 24 h (**c**), 3 days (**d**), and 1 week (**e**) post-LPS injection. Microglia appear to return to a surveying morphology at 2 weeks (**f**) post-LPS. *Scale bar* = 10 μM. **g**–**l** mRNA expression of inflammatory mediators in isolated cortical microglia from saline- and LPS-treated mice. Consistent with microglial morphologies, inflammatory markers are significantly upregulated by 6 h post-LPS injection. Maintenance of this pro-inflammatory phenotype is supported by the persistence of significantly elevated NOX2 expression which does not return to baseline levels until 1 week post-injection. Similarly, markers associated with resolution of inflammation are reduced following LPS injection, and expression of these markers does not return to control levels until 1 week post-injection. mRNA expression was evaluated by quantitative RT-PCR; values were normalized using the 2^−ΔΔCT^ method and were reported as mean expression ± SEM. An *asterisk* indicates significant difference (*p* < 0.05) from saline, and a ¥ indicates a difference between time points post-LPS injection

Previous studies demonstrated that chronic neuroinflammation in EAE resulted in disruption of the AIS, and this disruption coincided with microglial reactivity and increased microglial-AIS contact [17]. Therefore, to better understand how microglial inflammation contributes to AIS disruption in LPS-induced neuroinflammation, we examined microglial reactivity and gene expression of inflammatory mediators in saline- and LPS-treated mice. AIS disruption was first observed 24 h post-LPS injection and remained significantly disrupted until recovery 2 weeks post-injection. Therefore, we assessed microglial reactivity 6 h, 24 h, 3 days, 1 week, and 2 weeks post-injection. Iba-1 immunolabeling revealed that at 6 h post-LPS injection, prior to AIS changes, microglia display a reactive phenotype which is maintained 1 week post-LPS injection (Fig. 2b–e). By 2 weeks post-LPS injection (Fig. 2f), microglia morphology returned to a surveying phenotype similar to that of saline-injected mice (Fig. 2a). qPCR analysis of isolated microglia from LPS- and saline-injected mice revealed that gene expression of inflammatory mediators Tnf-α, COX-2, and NOX2 was significantly upregulated 6 h post-LPS injection (*p* < 0.05, Fig. 2g–i). Messenger RNA (mRNA) expression of NOX2, the enzyme responsible for extracellular release of ROS and amplification of microglial pro-inflammatory response [38], was significantly increased at 6 h (*p* < 0.01) and remained elevated 3 days post-LPS injection (*p* < 0.05), returning to control levels prior to AIS recovery (Fig. 2i). Furthermore, gene expression of resolving factors Mrc1, TGF-β, and Fizz-1 was significantly decreased by 24 h post-LPS injection and returned to control levels by 1 week post-injection, coincident with AIS disruption and recovery, respectively (*p* < 0.05, Fig. 2j–l). Thus, microglial inflammation preceded disruption of the AIS, while AIS recovery followed the resolution of microglial inflammation.

### Treatment with anti-inflammatory Didox reverses AIS disruption

**Fig. 3:**
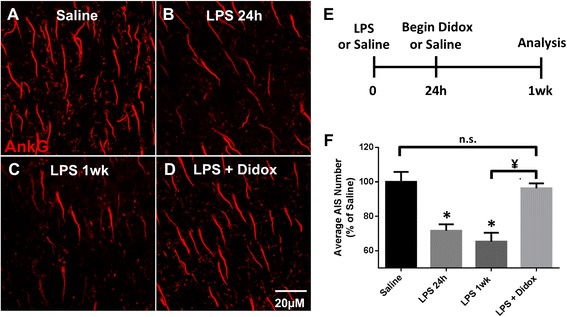
Treatment with Didox reverses AIS disruption. **a**–**d** AISs, immunolabeled for ankG, are reduced through 1 week post-injection. LPS-injected mice that received Didox treatment beginning at 24 h post-injection and continued for five subsequent days displayed no loss in ankG+-labeled AISs (**d**). *Scale bar* = 20 μM. **e** Schematic of LPS and anti-inflammatory Didox administration. **f** The mean ± SEM of AISs/FOV in saline-, LPS, and LPS + Didox-treated mice as a percent of saline controls. An *asterisk* indicates significant difference (*p* < 0.05) from saline, and a ¥ indicates a difference between treatment groups

In LPS-induced inflammation, microglia rapidly respond, displaying dramatic morphological alterations and significantly increasing expression of pro-inflammatory genes while significantly downregulating expression of pro-resolution factors (Fig. 2). mRNA expression of microglial inflammatory markers remained elevated and was not resolved until 1 week post-LPS injection. However, AIS clustering remained disrupted until 2 weeks post-LPS injection (Fig. 1). To determine if AIS recovery could be accelerated by a therapeutic approach, we treated LPS-injected mice with the anti-inflammatory and free radical scavenger Didox [47, 63–65]. Didox administration was initiated 24 h post-LPS injection (Fig. 3e), when microglia were reactive and AISs were significantly disrupted. Following treatment of saline- and LPS- injected mice, AISs were immunolabeled for ankG at 24 h and 1 week post-injection. At 24 h post-injection, ankG clustering in LPS-treated mice (71.6% ± 3.7, *p* < 0.01, Fig. 3b, f) was significantly disrupted compared to saline controls. The number of AISs in LPS-injected mice remained significantly decreased 1 week post-injection (65.3% ± 5.1, *p* < 0.001, Fig. 3c, f) compared to saline controls. However, in LPS + Didox mice, the number of AISs was significantly higher compared to that in LPS 1 week untreated mice (96.3% ± 2.8, *p* < 0.01) and was not significantly different from saline controls (Fig. 3d, f). Thus, treatment with Didox reversed AIS disruption and increased the rate of AIS recovery.

### Treatment with Didox alters microglial NOX2

**Fig. 4:**
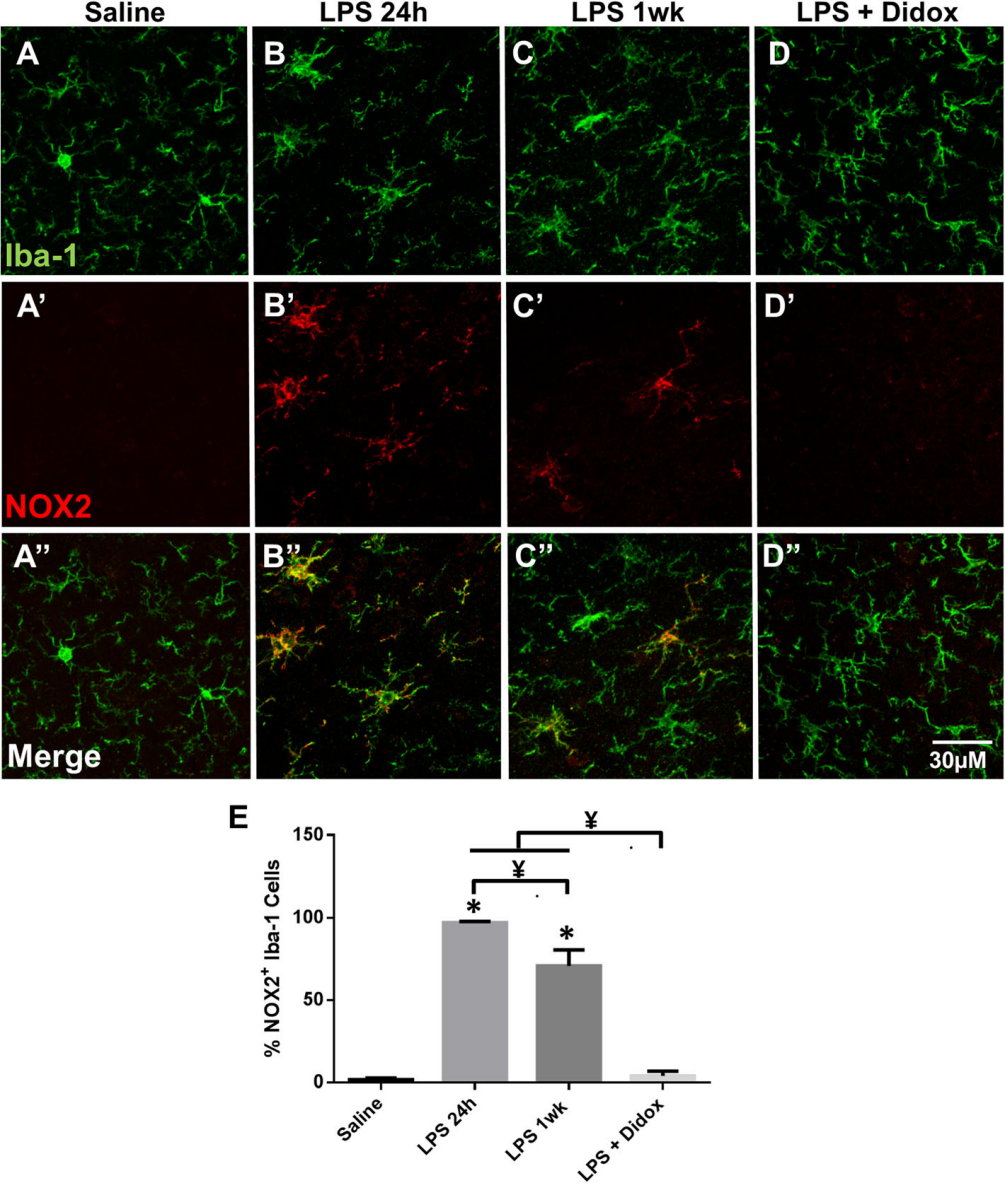
Treatment with Didox alters microglial NOX2 levels. **a**–**d** Microglia, visualized by Iba-1 immunolabeling, display enhanced NOX2 immunoreactivity in LPS 24 h (**b**’) and LPS 1 week (**c**’) mice compared to saline controls (**a**’). Anti-inflammatory Didox treatment decreases microglial NOX2 immunoreactivity (**d**’) to saline control levels (**a**’). *Scale bar* = 30 μM. **e** The mean percentage ± SEM of NOX2-positive microglia in saline-, LPS-treated, and LPS + Didox mice. An asterisk indicates significant difference (*p* < 0.05) from saline, and a ¥ indicates a difference between treatment groups

Didox is a ribonucleotide reductase inhibitor which modulates the inflammatory response through inhibition of NF-κβ activation, reduction in ROS-producing enzymes, and reduction in oxidative injury [47, 49, 66]. To determine the effect of the treatment with the anti-inflammatory and free radical scavenger Didox on microglial inflammatory response and AIS integrity following LPS treatment, we analyzed microglial NOX2, which is dependent on, and induced by, NF-κβ [67, 68]. NOX2 is a ROS-producing enzyme primarily expressed by microglia and has been implicated as the primary producer of extracellular ROS and oxidative stress in the CNS [39, 40, 69, 70]. Quantitation of NOX2 immunolabeling (Fig. 4A–D) in cortical microglia from saline- and LPS-treated mice with or without Didox treatment revealed that NOX2 immunoreactivity was significantly enhanced in microglia 24 h post-LPS injection (Fig. 4B, E) and remained significantly elevated 1 week post-LPS injection (Fig. 4C, E) compared to saline controls (*p* < 0.0001, Fig. 4A, E). However, NOX2 immunoreactivity was decreased in LPS-injected mice treated with the anti-inflammatory and free radical scavenger Didox (*p* < 0.0001, Fig. 4D, E). Thus, Didox treatment significantly decreased microglial NOX2 back to saline control levels, and this decrease corresponded with the reversal of AIS disruption.

### Ablation of NOX2-derived ROS production prevents AIS disruption

**Fig. 5:**
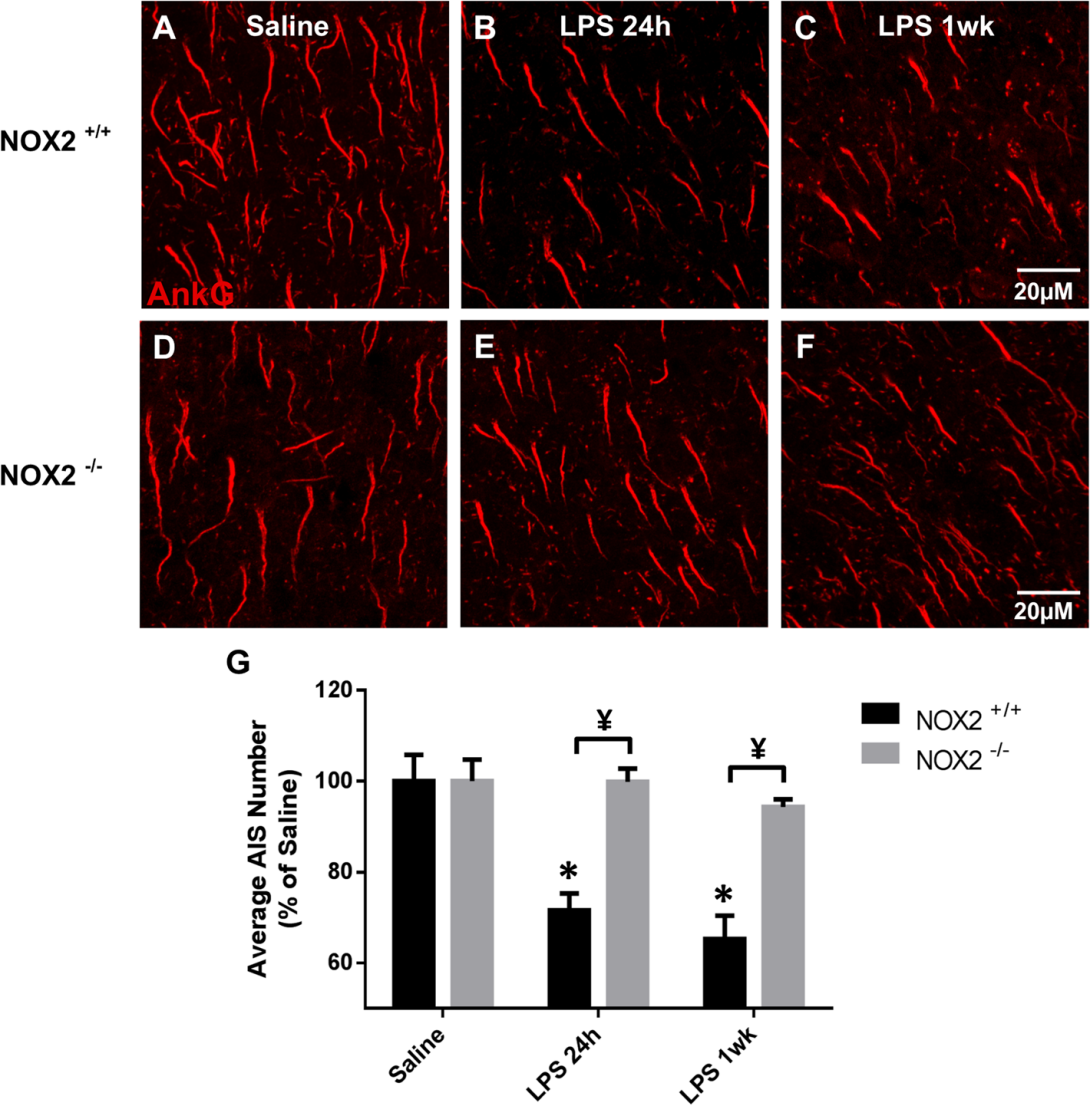
Ablation of NOX2-derived ROS production prevents AIS disruption. **a**–**f** AISs in layer V of the cerebral cortex from NOX2^+/+^ (**a**–**c**) and NOX2^−/−^ (**d**–**f**) mice are identified by ankG immunolabeling after saline (**a**, **d**) or LPS (**b**, **c**, **e**, **f**) treatment. AISs are lost in LPS-treated NOX2^+/+^ mice, but are preserved in LPS-treated NOX2^−/−^ mice. *Scale bar* = 20 μM. **g** The mean ± SEM of AISs/FOV in NOX2^+/+^ and NOX2^−/−^ mice treated with saline or LPS as a percent of saline controls. An *asterisk* indicates significant difference (*p* < 0.05) from saline, and a ¥ indicates a difference between genotypes

Although Didox is a known free radical scavenger and our data demonstrate a reduction in NOX2 expression resulting from Didox treatment, it is possible that Didox targets other inflammatory factors and that the observed AIS recovery was coincidental with inhibition of NOX2 expression. Therefore, to more specifically investigate the role that NOX2 plays in AIS disruption, we exploited NOX2^−/−^ mice. We injected NOX2^+/+^ and NOX2^−/−^ mice with saline or LPS and assessed AIS integrity 24 h and 1 week post-injection. ankG clustering in NOX2^+/+^ LPS-injected mice was significantly disrupted at 24 h (Fig. 5b) and 1 week (Fig. 5c) post-injection resulting in an ~30% loss of AISs, compared to saline controls (*p* < 0.01, Fig. 5g). Strikingly, ankG clustering in LPS-injected NOX2^−/−^ mice was not significantly different at either 24 h or 1 week post-injection compared to saline-injected NOX2^−/−^ mice (Fig. 5e, f). However, ankG clustering was significantly higher at both 24 h and 1 week compared to NOX2^+/+^ LPS-injected mice (*p* < 0.01, Fig. 5g). Thus, ablation of NOX2-derived ROS prevented inflammation-induced AIS disruption.

### Inhibition of calpain prevents AIS disruption

**Fig. 6:**
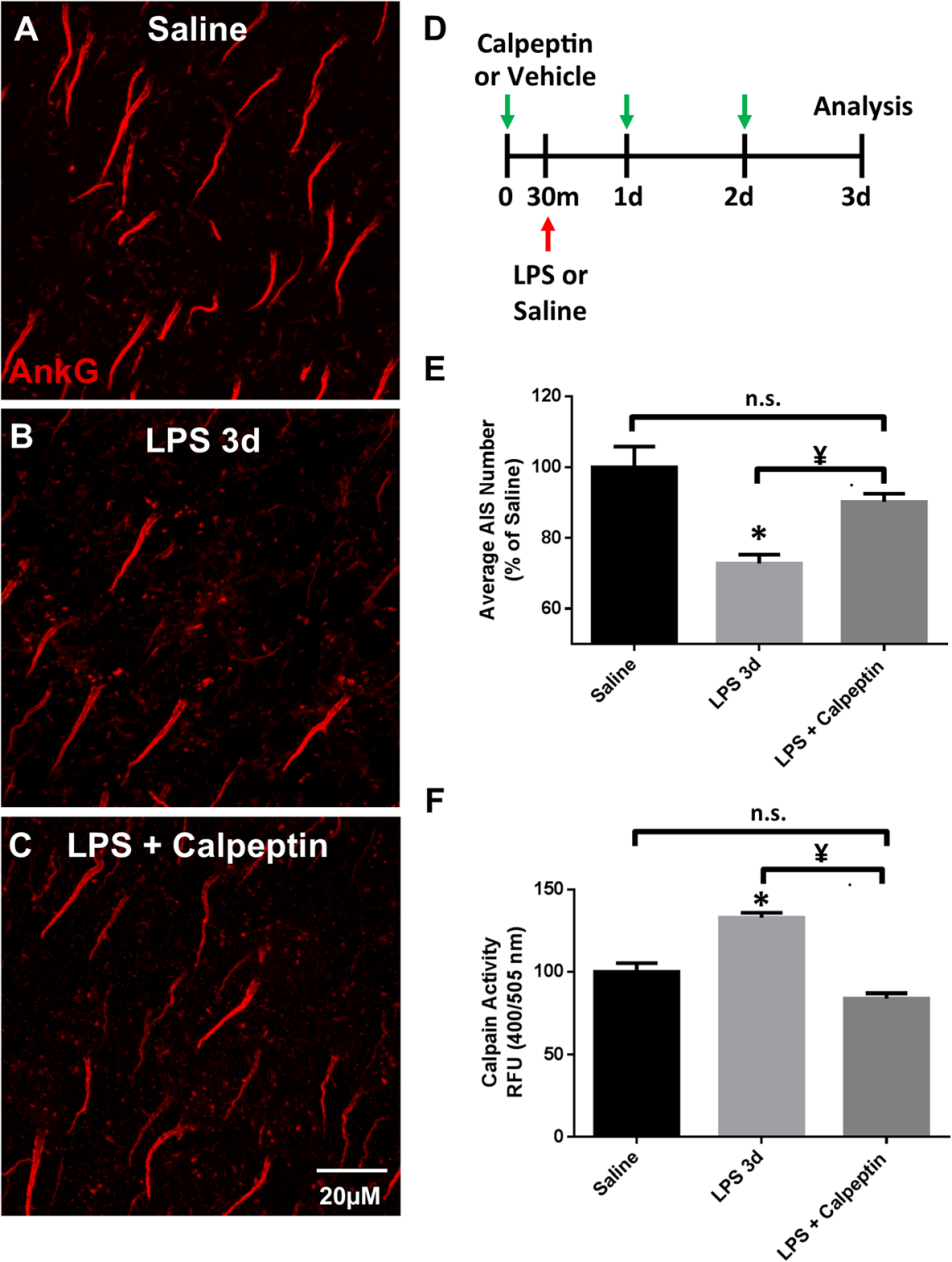
Treatment with calpain inhibitor prevents AIS disruption. **a**–**c** AISs, immunolabeled for ankG, are reduced 3 days post-LPS injection (**b**, **e**). LPS-injected mice that received Calpeptin treatment (calpain inhibitor) beginning 30 min prior to LPS injection and continued once daily for 2 days (**d**) displayed no loss in ankG^+^-labeled AISs (**c**, **e**). *Scale bar* = 20 μM. **d** Schematic of LPS and Calpeptin administration. **e** The mean ± SEM of AISs/FOV in saline-, LPS + vehicle-, and LPS + Calpeptin-treated mice as a percent of saline controls. **f** The mean ± SEM of calpain activity levels in relative fluorescent units in saline-, LPS + vehicle-, and LPS + Calpeptin-treated mice as a percent of saline controls. An *asterisk* indicates significant difference (*p* < 0.05) from saline, and a ¥ indicates a difference between treatment groups

To further elucidate the mechanism of inflammation-induced AIS disruption, we investigated the calcium-activated protease calpain. Calpain activity has been implicated in AIS structural changes and the targeted proteolysis of AIS proteins [9, 10, 14]. To determine if calpain activity is involved in inflammation-induced loss of AIS ankG clustering, we treated LPS-injected mice with the calpain inhibitor Calpeptin. Calpeptin administration was initiated 30 min prior to LPS injection and continued once daily for 2 days (Fig. 6d). Following treatment, AISs were immunolabeled for ankG at 3 days post-LPS injection. ankG clustering in LPS-treated mice (72.7% ± 2.5, *p* < 0.01, Fig. 6b, e) was significantly disrupted compared to saline controls (100% ± 5.8, Fig. 6a, e). However, in LPS + Calpeptin-treated mice, the number of AISs was significantly higher compared to that in LPS 3 days vehicle-treated mice (90.2% ± 2.3 and 72.8% ± 2.5, respectively, *p* < 0.05, Fig. 6c, e) and was not significantly different from saline controls. Inhibition of calpain activity by Calpeptin was determined by a fluorometric calpain activity assay on mouse cortical homogenates 3 days post-LPS injection (Fig. 6f). Calpain activity was significantly increased in LPS 3 days mice (132.9% ± 3.0, *p* < 0.01, Fig. 6f) compared to saline controls (100% ± 5.3), and treatment with Calpeptin significantly reduced calpain activity in cortical homogenates (84.1% ± 3.1, *p* < 0.001, Fig. 6e). Thus, inhibition of calpain activity prevents inflammation-induced disruption of AIS ankG clustering in cortical neurons.

## Discussion

In this study, we demonstrate that LPS-induced neuroinflammation disrupts protein clustering at the AIS concomitant with the microglial inflammatory response resulting in an ~30% loss of AIS detection. Importantly, we found that inflammation-induced AIS disruptions were reversed following resolution of microglial inflammation and changes in AIS ankG clustering are NOX2-mediated and dependent on calpain activity. Thus, in the presence of acute microglial inflammation, the AIS undergoes an adaptive change that is capable of spontaneous recovery, underscoring the dynamic capabilities of this domain in the presence of a pathological insult.

### The AIS has the capacity to adapt and recover

The AIS is targeted for disruption in injury and disease emphasizing its need for homeostatic adaptations. Indeed, many studies [16, 17, 37, 71–73] have shown that the AIS is plastic, undergoing change in response to various stimuli. However, few studies have demonstrated that these changes are reversible. Alterations in AIS length [12] and location [2] caused by changes in neural activity were reversible in vitro; however, loss of AIS protein clustering due to ischemic insults in vitro were not, even in the absence of cell death [14]. A previous study examining AIS integrity after stroke observed axonal sprouting resulting in an increase in small, immature AISs demonstrating reparative potential of this domain [59]. Furthermore, our lab previously reported that shortening of AIS length is reversible following treatment with the anti-inflammatory Didox [17]. Here, we provide evidence that loss of AIS protein clustering is spontaneously reversible, independent of axonal sprouting. Moreover, we show that by modulating the neuroinflammatory response using therapeutic intervention, the rate of AIS recovery can be increased, even after significant AIS disruption has occurred. These data suggest that while insults at the AIS, such as ischemia [14], can cause irreversible damage, the AIS has the capacity to adapt and recover after insult. The mechanism by which this occurs or what the extent of injury is after which the AIS cannot recover remains to be determined.

### Microglial phenotype influences AIS integrity

Although AIS plasticity can be triggered by both pathological and non-pathological stimuli, the events that drive plasticity remain largely unknown. Recently, Baalman et al. [16] established a relationship between microglia and the AIS, revealing that microglia contact AISs early in development and throughout adulthood in the uninjured brain, suggesting an important interaction that may influence neuronal excitability. In a model of chronic neuroinflammation, reactive microglia increased contact with AISs, and this contact both preceded AIS disruption and increased with disease progression, suggesting that in a chronic inflammatory environment, increased microglial contact may drive AIS disruptions [17]. Consistent with previous findings, we found that reactive microglia contact the AISs during LPS-induced neuroinflammation. However, contrary to findings in the chronic inflammatory model, the amount of contact made by microglia did not increase throughout the course of inflammation and did not correlate with AIS disruption (data not shown). The microglial inflammatory profile, however, did correspond with AIS disruption and recovery. Furthermore, modulation of the inflammatory profile using anti-inflammatory treatment increased the rate of AIS recovery. Though our findings suggest that changes in microglial inflammation correspond with AIS alterations, it is possible that these changes do not directly influence AIS integrity. However, the direct association of microglia with the AIS suggests this axonal domain may be particularly vulnerable to changes in microglial reactivity. Thus, our findings suggest that AIS integrity may be influenced by microglial phenotype, with a pro-inflammatory phenotype driving AIS disruption while a resolving phenotype hastens repair.

Consistent with this premise, Klapal et al. [74] showed that incubation of hippocampal cultures with activated microglia or the pro-inflammatory cytokine Tnf-α increased neuronal excitability. In contrast, incubation with the pro-resolution factor TGF-β decreased Na^+^ current density to control levels. Together, these findings suggest that neuroactive factors released by microglia augment neuronal excitability, which drives AIS structural changes [2, 6, 12]. Here, we demonstrate that AIS structure is altered following significant increases in microglial expression of Tnf-α. Furthermore, this AIS pathology is reversed after expression of microglial TGF-β is enhanced. Thus, our findings are consistent with microglial neuroactive factors driving changes in neuronal activity and AIS structural plasticity.

### NOX2-mediated ROS, calpain, and AIS changes

During insult, pro-inflammatory microglia increase expression of inflammatory mediators and ROS-producing enzymes [30, 40, 45, 75]. ROS are highly reactive and diffuse signaling molecules that regulate cell functions through redox modification of target proteins. ROS can result in further production of reactive species [76, 77] and elevated calcium levels [78], which have been implicated in AIS disruption. In this study, we show that changes in microglial expression of ROS-producing enzymes correspond with AIS disruption and recovery, suggesting a role for microglial ROS in inflammation-driven AIS disruption. Consistent with this premise, ablation of NOX2 prevented AIS disruption. Though NOX2 is primarily expressed by microglia, NOX2 is also present in cortical neurons, where it plays a role in ROS regulation and calcium dynamics [79]. Therefore, NOX2 ablation may preserve AIS integrity through both the prevention of microglial ROS release and neuronal NOX2 ROS production, both of which may converge on pathways resulting in AIS changes.

Reactive species such as hydrogen peroxide and nitric oxide influence calcium-permeable channels including L-type Ca(^2+^) [78, 80, 81] and TRPM channels [82, 83]. Upon activation, intracellular calcium concentrations rise, resulting in the subsequent activation of calcium-regulated proteins such as calpain [9, 10, 14], CamKII [8], and calcineurin [8, 12], which have been implicated in AIS disruption. Consistent with previous studies [9, 10, 14], our data implicate the calcium-dependent protease calpain as a mediator of AIS structural changes. In this study, we demonstrate that acute neuroinflammation increases calpain activity consistent with disruption of AIS ankG clustering and inhibition of calpain prevents inflammation-induced disruptions. Together, our data suggest that NOX2-derived ROS and calpain activity are drivers of AIS structural plasticity during acute neuroinflammation.

## Conclusions

In conclusion, we demonstrate that in the presence of acute neuroinflammation, protein clustering at the AIS is altered. Importantly, our data demonstrate that this AIS disruption is reversible and that the AIS has the capacity to adapt and spontaneously recover. Furthermore, we reveal that inflammation-driven plasticity at the AIS is mediated by NOX2 and calpain activity.

## Abbreviations

AIS: Axon initial segment
ankG: AnkyrinG
CNS: Central nervous system
COX-2: Cyclooxygenase-2
EAE: Experimental autoimmune encephalomyelitis
Fizz-1 (Retnlb): Resistin-like beta
HBSS: Hank’s balanced salt solution
Iba-1: Ionized calcium-binding adapter molecule 1
LPS: Lipopolysaccharide
Mrc1: Mannose receptor, C type 1
NaV1.6: Voltage-gated sodium channel 1.6
NeuN: Neuronal nuclei, Rbfox-3
NOX2: NADPH oxidase 2, gp91 phox
PGK1: Phosphoglycerate kinase 1
ROS: Reactive oxygen species
TGF-β: Transforming growth factor beta
Tnf-α: Tumor necrosis factor alpha

## References

[1] Yoshimura T, Rasband MN (2014). Axon initial segments: diverse and dynamic neuronal compartments. Curr Opin Neurobiol.

[2] Grubb MS, Burrone J (2010). Activity-dependent relocation of the axon initial segment fine-tunes neuronal excitability. Nature.

[3] Yang Y, Ogawa Y, Hedstrom KL, Rasband MN (2007). betaIV spectrin is recruited to axon initial segments and nodes of Ranvier by ankyrinG. J Cell Biol.

[4] Hedstrom KL, Ogawa Y, Rasband MN (2008). AnkyrinG is required for maintenance of the axon initial segment and neuronal polarity. J Cell Biol.

[5] Zhou D, Lambert S, Malen PL, Carpenter S, Boland LM, Bennett V (1998). AnkyrinG is required for clustering of voltage-gated Na channels at axon initial segments and for normal action potential firing. J Cell Biol.

[6] Kuba H, Oichi Y, Ohmori H (2010). Presynaptic activity regulates Na(+) channel distribution at the axon initial segment. Nature.

[7] Wefelmeyer W, Cattaert D, Burrone J (2015). Activity-dependent mismatch between axo-axonic synapses and the axon initial segment controls neuronal output. Proc Natl Acad Sci U S A.

[8] Evans MD, Sammons RP, Lebron S, Dumitrescu AS, Watkins TBK, Uebele VN (2013). Calcineurin signaling mediates activity-dependent relocation of the axon initial segment. J Neurosci Off J Soc Neurosci.

[9] Benned-Jensen T, Christensen RK, Denti F, Perrier J-F, Rasmussen HB, Olesen S-P (2016). Live imaging of Kv7.2/7.3 cell surface dynamics at the axon initial segment: high steady-state stability and calpain-dependent excitotoxic downregulation revealed. J Neurosci Off J Soc Neurosci.

[10] Del Puerto A, Fronzaroli-Molinieres L, Perez-Alvarez MJ, Giraud P, Carlier E, Wandosell F (2015). ATP-P2X7 receptor modulates axon initial segment composition and function in physiological conditions and brain injury. Cereb Cortex N Y N 1991.

[11] Chand AN, Galliano E, Chesters RA, Grubb MS (2015). A distinct subtype of dopaminergic interneuron displays inverted structural plasticity at the axon initial segment. J Neurosci Off J Soc Neurosci.

[12] Evans MD, Dumitrescu AS, Kruijssen DLH, Taylor SE, Grubb MS (2015). Rapid modulation of axon initial segment length influences repetitive spike firing. Cell Rep.

[13] Muir J, Kittler JT (2014). Plasticity of GABAA receptor diffusion dynamics at the axon initial segment. Front Cell Neurosci.

[14] Schafer DP, Jha S, Liu F, Akella T, McCullough LD, Rasband MN (2009). Disruption of the axon initial segment cytoskeleton is a new mechanism for neuronal injury. J Neurosci Off J Soc Neurosci.

[15] Stoler O, Fleidervish IA (2016). Functional implications of axon initial segment cytoskeletal disruption in stroke. Acta Pharmacol Sin.

[16] Baalman K, Marin MA, Ho TS-Y, Godoy M, Cherian L, Robertson C (2015). Axon initial segment-associated microglia. J Neurosci Off J Soc Neurosci.

[17] Clark KC, Josephson A, Benusa SD, Hartley RK, Baer M, Thummala S (2016). Compromised axon initial segment integrity in EAE is preceded by microglial reactivity and contact. Glia.

[18] Crotti A, Ransohoff RM (2016). Microglial physiology and pathophysiology: insights from genome-wide transcriptional profiling. Immunity.

[19] Kettenmann H, Kirchhoff F, Verkhratsky A (2013). Microglia: new roles for the synaptic stripper. Neuron.

[20] Miyamoto A, Wake H, Ishikawa AW, Eto K, Shibata K, Murakoshi H (2016). Microglia contact induces synapse formation in developing somatosensory cortex. Nat Commun.

[21] Schafer DP, Stevens B (2013). Phagocytic glial cells: sculpting synaptic circuits in the developing nervous system. Curr Opin Neurobiol.

[22] Kierdorf K, Prinz M (2013). Factors regulating microglia activation. Front Cell Neurosci.

[23] Ransohoff RM, Perry VH (2009). Microglial physiology: unique stimuli, specialized responses. Annu Rev Immunol.

[24] Tremblay M-È, Stevens B, Sierra A, Wake H, Bessis A, Nimmerjahn A (2011). The role of microglia in the healthy brain. J Neurosci.

[25] Aguzzi A, Barres BA, Bennett ML (2013). Microglia: scapegoat, saboteur, or something else?. Science.

[26] Prinz M, Tay TL, Wolf Y, Jung S (2014). Microglia: unique and common features with other tissue macrophages. Acta Neuropathol (Berl).

[27] Wong WT (2013). Microglial aging in the healthy CNS: phenotypes, drivers, and rejuvenation. Front Cell Neurosci.

[28] Sierra A, Abiega O, Shahraz A, Neumann H (2013). Janus-faced microglia: beneficial and detrimental consequences of microglial phagocytosis. Front Cell Neurosci.

[29] Cherry JD, Olschowka JA, O’Banion MK (2014). Neuroinflammation and M2 microglia: the good, the bad, and the inflamed. J Neuroinflammation.

[30] Block ML, Zecca L, Hong J-S (2007). Microglia-mediated neurotoxicity: uncovering the molecular mechanisms. Nat Rev Neurosci.

[31] Ohl K, Tenbrock K, Kipp M (2016). Oxidative stress in multiple sclerosis: central and peripheral mode of action. Exp Neurol.

[32] Gomez-Nicola D, Perry VH (2015). Microglial dynamics and role in the healthy and diseased brain: a paradigm of functional plasticity. Neurosci Rev J Bringing Neurobiol Neurol Psychiatry.

[33] Edison P, Archer HA, Gerhard A, Hinz R, Pavese N, Turkheimer FE (2008). Microglia, amyloid, and cognition in Alzheimer’s disease: an [11C](R)PK11195-PET and [11C]PIB-PET study. Neurobiol Dis.

[34] Russo MV, McGavern DB (2016). Inflammatory neuroprotection following traumatic brain injury. Science.

[35] Johnson VE, Stewart JE, Begbie FD, Trojanowski JQ, Smith DH, Stewart W (2013). Inflammation and white matter degeneration persist for years after a single traumatic brain injury. Brain.

[36] Marin MA, Ziburkus J, Jankowsky J, Rasband MN (2016). Amyloid-β plaques disrupt axon initial segments. Exp Neurol.

[37] Baalman KL, Cotton RJ, Rasband SN, Rasband MN (2013). Blast wave exposure impairs memory and decreases axon initial segment length. J Neurotrauma.

[38] Wang Z, Wei X, Liu K, Zhang X, Yang F, Zhang H (2013). NOX2 deficiency ameliorates cerebral injury through reduction of complexin II-mediated glutamate excitotoxicity in experimental stroke. Free Radic Biol Med.

[39] Kumar A, Barrett JP, Alvarez-Croda D-M, Stoica BA, Faden AI, Loane DJ. NOX2 drives M1-like microglial/macrophage activation and neurodegeneration following experimental traumatic brain injury. Brain Behav Immun. doi:10.1016/j.bbi.2016.07.158. 10.1016/j.bbi.2016.07.158 PMC5067217 27477920

[40] Qin L, Liu Y, Wang T, Wei S-J, Block ML, Wilson B (2004). NADPH oxidase mediates lipopolysaccharide-induced neurotoxicity and proinflammatory gene expression in activated microglia. J Biol Chem.

[41] Pawate S, Shen Q, Fan F, Bhat NR (2004). Redox regulation of glial inflammatory response to lipopolysaccharide and interferongamma. J Neurosci Res.

[42] Choi DC, Lee JY, Lim EJ, Baik HH, Oh TH, Yune TY (2012). Inhibition of ROS-induced p38MAPK and ERK activation in microglia by acupuncture relieves neuropathic pain after spinal cord injury in rats. Exp Neurol.

[43] Qin L, Liu Y, Hong J-S, Crews FT (2013). NADPH oxidase and aging drive microglial activation, oxidative stress, and dopaminergic neurodegeneration following systemic LPS administration. Glia.

[44] Pollock JD, Williams DA, Gifford MA, Li LL, Du X, Fisherman J (1995). Mouse model of X-linked chronic granulomatous disease, an inherited defect in phagocyte superoxide production. Nat Genet.

[45] Taetzsch T, Levesque S, McGraw C, Brookins S, Luqa R, Bonini MG (2015). Redox regulation of NF-κB p50 and M1 polarization in microglia. Glia.

[46] Qin L, Wu X, Block ML, Liu Y, Breese GR, Hong J-S (2007). Systemic LPS causes chronic neuroinflammation and progressive neurodegeneration. Glia.

[47] Matsebatlela TM, Anderson AL, Gallicchio VS, Elford H, Rice CD (2015). 3,4-Dihydroxy-benzohydroxamic acid (Didox) suppresses pro-inflammatory profiles and oxidative stress in TLR4-activated RAW264.7 murine macrophages. Chem Biol Interact.

[48] Shah KN, Wilson EA, Malla R, Elford HL, Faridi JS (2015). Targeting ribonucleotide reductase M2 and NF-κB activation with Didox to circumvent tamoxifen resistance in breast cancer. Mol Cancer Ther.

[49] Turchan J, Pocernich CB, Gairola C, Chauhan A, Schifitto G, Butterfield DA (2003). Oxidative stress in HIV demented patients and protection ex vivo with novel antioxidants. Neurology.

[50] DeVries GH, Farrer R, Papadopoulos C, Campbell C, Litz J, Paletta J (2012). Didox—a multipotent drug for treating demyelinating disease. FASEB J.

[51] Guyton MK, Das A, Samantaray S, Wallace GC, Butler JT, Ray SK (2010). Calpeptin attenuated inflammation, cell death, and axonal damage in animal model of multiple sclerosis. J Neurosci Res.

[52] Smith AW, Das A, Guyton MK, Ray SK, Rohrer B, Banik NL (2011). Calpain inhibition attenuates apoptosis of retinal ganglion cells in acute optic neuritis. Invest Ophthalmol Vis Sci.

[53] Das A, Guyton MK, Smith A, Wallace G, McDowell ML, Matzelle DD (2013). Calpain inhibitor attenuated optic nerve damage in acute optic neuritis in rats. J Neurochem.

[54] Otsu N (1979). A Threshold Selection Method from Gray-Level Histograms. IEEE Transactions on Systems, Man, and Cybernetics..

[55] Hahn YK, Podhaizer EM, Farris SP, Miles MF, Hauser KF, Knapp PE (2015). Effects of chronic HIV-1 Tat exposure in the CNS: heightened vulnerability of males versus females to changes in cell numbers, synaptic integrity, and behavior. Brain Struct Funct.

[56] Mouton PR. Neurostereology: unbiased stereology of neural systems. Hoboken, NJ: Wiley; 2013.

[57] Livak KJ, Schmittgen TD (2001). Analysis of relative gene expression data using real-time quantitative PCR and the 2(-delta delta C(T)) method. Methods San Diego Calif.

[58] Ye J, Coulouris G, Zaretskaya I, Cutcutache I, Rozen S, Madden TL (2012). Primer-BLAST: a tool to design target-specific primers for polymerase chain reaction. BMC Bioinformatics.

[59] Hinman JD, Rasband MN, Carmichael ST (2013). Remodeling of the axon initial segment after focal cortical and white matter stroke. Stroke.

[60] Buffington SA, Rasband MN (2011). The axon initial segment in nervous system disease and injury. Eur J Neurosci.

[61] Kuba H, Yamada R, Ishiguro G, Adachi R (2015). Redistribution of Kv1 and Kv7 enhances neuronal excitability during structural axon initial segment plasticity. Nat Commun.

[62] Gutzmann A, Ergül N, Grossmann R, Schultz C, Wahle P, Engelhardt M (2014). A period of structural plasticity at the axon initial segment in developing visual cortex. Front Neuroanat.

[63] Inayat MS, El-Amouri IS, Bani-Ahmad M, Elford HL, Gallicchio VS, Oakley OR (2010). Inhibition of allogeneic inflammatory responses by the ribonucleotide reductase inhibitors, Didox and Trimidox. J Inflamm Lond Engl.

[64] Inayat MS, Chendil D, Mohiuddin M, Elford HL, Gallicchio VS, Ahmed MM (2002). Didox (a novel ribonucleotide reductase inhibitor) overcomes Bcl-2 mediated radiation resistance in prostate cancer cell line PC-3. Cancer Biol Ther.

[65] Mayhew CN, Mampuru LJ, Chendil D, Ahmed MM, Phillips JD, Greenberg RN (2002). Suppression of retrovirus-induced immunodeficiency disease (murine AIDS) by trimidox and didox: novel ribonucleotide reductase inhibitors with less bone marrow toxicity than hydroxyurea. Antiviral Res.

[66] Elford H, Lee R, Turchan J, Gallicchio V, Ussery M, Hiscott J (2007). The virtues of unique ribonucleotide reductase inhibitors Didox and Trimidox for retrovirus therapy. Antiviral Res.

[67] Anrather J, Racchumi G, Iadecola C (2006). NF-kappaB regulates phagocytic NADPH oxidase by inducing the expression of gp91phox. J Biol Chem.

[68] Morgan MJ, Liu Z (2011). Crosstalk of reactive oxygen species and NF-κB signaling. Cell Res.

[69] Nayernia Z, Jaquet V, Krause K-H (2014). New insights on NOX enzymes in the central nervous system. Antioxid Redox Signal.

[70] Guemez-Gamboa A, Estrada-Sánchez AM, Montiel T, Páramo B, Massieu L, Morán J (2011). Activation of NOX2 by the stimulation of ionotropic and metabotropic glutamate receptors contributes to glutamate neurotoxicity in vivo through the production of reactive oxygen species and calpain activation. J Neuropathol Exp Neurol.

[71] Hamada MS, Kole MHP (2015). Myelin loss and axonal ion channel adaptations associated with gray matter neuronal hyperexcitability. J Neurosci Off J Soc Neurosci.

[72] Harty RC, Kim TH, Thomas EA, Cardamone L, Jones NC, Petrou S (2013). Axon initial segment structural plasticity in animal models of genetic and acquired epilepsy. Epilepsy Res.

[73] Kaphzan H, Buffington SA, Jung JI, Rasband MN, Klann E (2011). Alterations in intrinsic membrane properties and the axon initial segment in a mouse model of Angelman syndrome. J Neurosci Off J Soc Neurosci.

[74] Klapal L, Igelhorst BA, Dietzel-Meyer ID (2016). Changes in neuronal excitability by activated microglia: differential Na + current upregulation in pyramid-shaped and bipolar neurons by TNF-α and IL-18. Neurotrauma.

[75] Bordt EA, Polster BM (2014). NADPH oxidase- and mitochondria-derived reactive oxygen species in proinflammatory microglial activation: a bipartisan affair?. Free Radic Biol Med.

[76] Feissner RF, Skalska J, Gaum WE, Sheu S-S (2009). Crosstalk signaling between mitochondrial Ca2+ and ROS. Front Biosci Landmark Ed.

[77] Peng T-I, Jou M-J (2010). Oxidative stress caused by mitochondrial calcium overload. Ann N Y Acad Sci.

[78] Hudasek K, Brown ST, Fearon IM (2004). H2O2 regulates recombinant Ca2+ channel α1C subunits but does not mediate their sensitivity to acute hypoxia. Biochem Biophys Res Commun.

[79] Wang X, Pinto-Duarte A, Sejnowski TJ, Behrens MM (2013). How Nox2-containing NADPH oxidase affects cortical circuits in the NMDA receptor antagonist model of schizophrenia. Antioxid Redox Signal.

[80] Hool LC (2008). Evidence for the regulation of L-type Ca2+ channels in the heart by reactive oxygen species: mechanism for mediating pathology. Clin Exp Pharmacol Physiol.

[81] Hool LC, Arthur PG (2002). Decreasing cellular hydrogen peroxide with catalase mimics the effects of hypoxia on the sensitivity of the L-type Ca2+ channel to β-adrenergic receptor stimulation in cardiac myocytes. Circ Res.

[82] Wehage E, Eisfeld J, Heiner I, Jüngling E, Zitt C, Lückhoff A (2002). Activation of the cation channel long transient receptor potential channel 2 (LTRPC2) by hydrogen peroxide. A splice variant reveals a mode of activation independent of ADP-ribose. J Biol Chem.

[83] Aarts MM, Tymianski M (2005). TRPM7 and ischemic CNS injury. Neurosci Rev J Bringing Neurobiol Neurol Psychiatry.

